# Evaluating bias-reducing protocols for RNA sequencing library preparation

**DOI:** 10.1186/1471-2164-15-569

**Published:** 2014-07-07

**Authors:** Thomas J Jackson, Ruth V Spriggs, Nicholas J Burgoyne, Carolyn Jones, Anne E Willis

**Affiliations:** Medical Research Council Toxicology Unit, Lancaster Rd, Leicester, LE1 9HN UK; University of Nottingham Medical School, Queen’s Medical Centre, Nottingham, NG7 2UH UK

**Keywords:** Next-generation sequencing, Bias, Ion Torrent, Mth K97A, Thermostable, CircLigase, Small RNA

## Abstract

**Background:**

Next-generation sequencing does not yield fully unbiased estimates for read abundance, which may impact on the conclusions that can be drawn from sequencing data. The ligation step in RNA sequencing library generation is a known source of bias, motivating developments in enzyme technology and library construction protocols. We present the first comparison of the standard duplex adaptor protocol supplied by Life Technologies for use on the Ion Torrent PGM with an alternate single adaptor approach involving CircLigase (CircLig protocol).

A correlation between over-representation in sequenced libraries and degree of secondary structure has been reported previously, therefore we also investigated whether bias could be reduced by ligation with an enzyme that functions at a temperature not permissive for such structure.

**Results:**

A pool of small RNA fragments of known composition was converted into a sequencing library using one of three protocols and sequenced on an Ion Torrent PGM. The CircLig protocol resulted in less over-representation of specific sequences than the standard protocol. Over-represented sequences are more likely to be predicted to have secondary structure and to co-fold with adaptor sequences. However, use of the thermostable ligase *Methanobacterium thermoautotrophicum* RNA ligase K97A (Mth K97A) was not sufficient to reduce bias.

**Conclusions:**

The single adaptor CircLigase-based approach significantly reduces, but does not eliminate, bias in Ion Torrent data. Ligases that function at temperatures to remove the possible influence of secondary structure on library generation may be of value, although Mth K97A is not effective in this case.

**Electronic supplementary material:**

The online version of this article (doi:10.1186/1471-2164-15-569) contains supplementary material, which is available to authorized users.

## Background

Next-generation sequencing is a powerful genomic tool that can be used to investigate the transcriptome (the abundance of RNA species), the translatome (the ribosome occupancy on mRNAs) and the interactome (RNA-protein binding). However, substantial differences in data obtained have been observed when the same fragment library is sequenced on different platforms [1]. This has motivated attempts to characterise and remedy biases in sequencing library generation, either through modification of protocols [2, 3] or applying bioinformatic corrections [4].

Whilst there are a number of different sequencing platforms, each share a common series of steps to convert the RNA pool of interest into an RNA-seq library. Each platform has a specific set of adaptors that are ligated to both 5′ and 3′ ends of a pool of RNA fragments of interest (e.g. small RNAs or fragmented mRNAs). These adaptors are then used to prime reverse transcription and PCR amplification. These completed libraries are then sequenced, using the adaptors as the starting point for the sequencing reaction.

However, the biochemical manipulations involved in this library generation process introduce biases that affect the final sequencing output. PCR amplification results in under-representation of both AT rich and GC rich regions [5, 6], but this can be minimised by the use of polymerases that have been generated through molecular evolution to reduce these biases such as KAPA HiFi [7]. Single molecule studies have shown that T4 RNA ligases 1 and 2 (rnl1 and rnl2), which are used to ligate adaptor sequences, are also associated with significant biases (reviewed in [8]). A point mutation of the truncated rnl2 (trRnl2 K227Q) reduces bias [9], but fragments predicted to co-fold with the adaptor are still over-represented in sequencing libraries when standard protocols are used [10]. To address whether ligation at temperatures that minimise co-folding reduces bias, a thermostable 3′RNA ligase has been developed: *Methanobacterium thermoautotrophicum* RNA ligase K97A mutant (Mth K97A) [11]. To date, the suitability of Mth K97A for use in RNA-seq has not been assessed.

**Figure 1:**
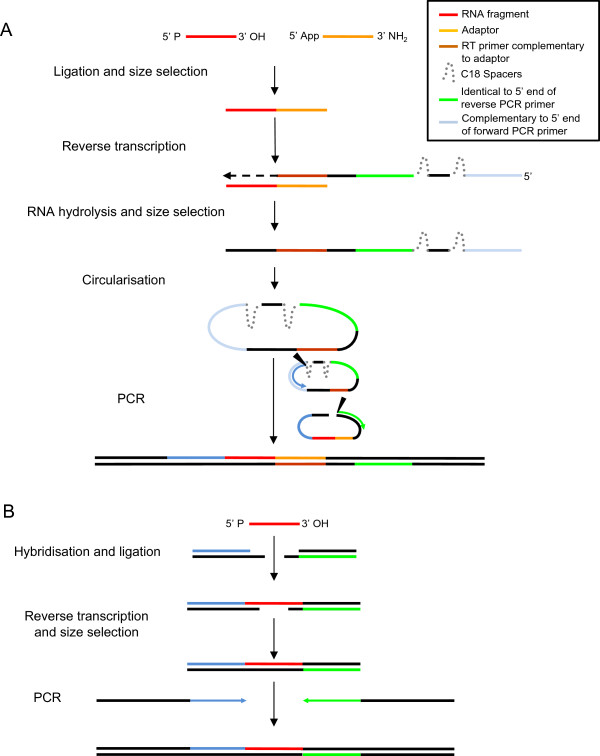
**Schematic of RNA-seq library generation protocols.** RNA fragments are ligated to adaptors that permit reverse transcription and PCR amplification prior to sequencing. **(A)** The CircLig protocol involves ligation to a ssDNA adaptor prior to reverse transcription with a primer that results in PCR primer sites either side of the fragment when the cDNA is circularised. **(B)** The Life Technologies protocol involves hybridisation to duplexed RNA-DNA adaptors containing degenerate ssDNA overhangs prior to ligation.

Alongside developments in enzyme technology, new ligation strategies have been developed. For example, the CircLig protocol avoids the use of rnl1. In this approach an adaptor is ligated to the 3′ end of fragments with trRnl2 K227Q and used to prime reverse transcription with a primer containing two PCR primer sites. The resulting cDNA is circularised resulting in PCR primer sites either side of the fragment permitting amplification [2] (Figure 1A). 

Library generation for Life Technologies’ sequencing platforms (SOLiD and Ion Torrent) employs hybridisation to duplex adaptors with degenerate overhangs prior to ligation with trRnl2 (Figure 1B).

Herein we present the first direct comparison of the CircLig and standard Life Technologies protocols. Furthermore, to investigate whether Mth K97A might be suitable for use in RNA-seq library generation, we substituted it for trRnl2 K227Q in the CircLig protocol. The degree of bias was assessed by generating libraries from a pool of 20 nt RNAs of known composition using each protocol and sequencing them on an Ion Torrent personal genome machine.

## Results

### Comparing over-representation introduced by different library preparation protocols

**Figure 2:**
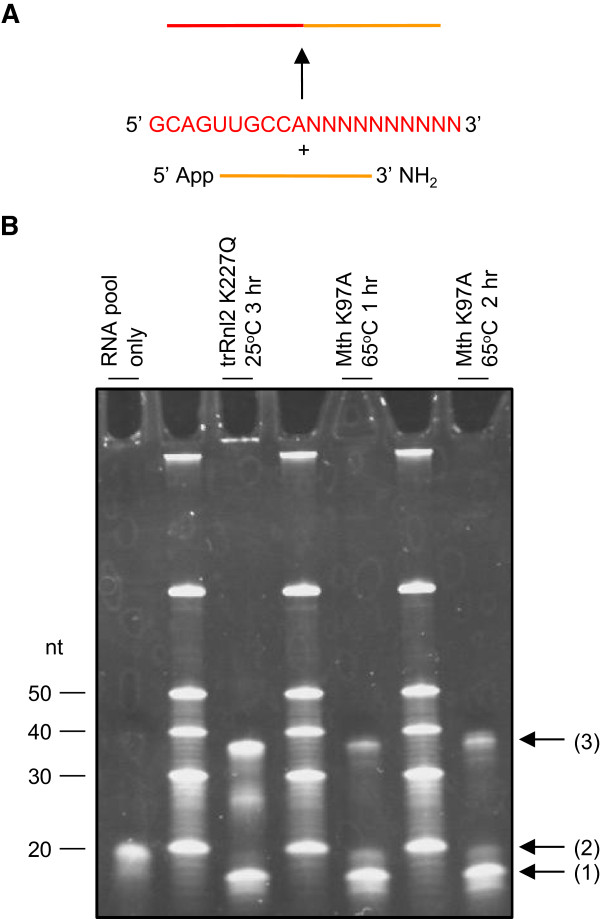
**Ligation of RNA pool to universal adaptor in CircLig protocol. (A)** Schematic of the ligation reaction. **(B)** 40 ng RNA pool was ligated in the presence of excess universal adaptor with either trRnl2 K227Q or Mth K97A as described in the Methods. RNA was precipitated and separated on a 15% acrylamide gel. (1) Universal adaptor (2) fragment pool (3) ligated fragments.

An RNA fragment pool composed of 10 nt chosen at random followed by 10 nt of degenerate sequence (5′ – GCAGUUGCCANNNNNNNNNN –3′) was converted into a cDNA sequencing library using either the standard Ion Torrent protocol (standard), CircLig protocol with trRnl2 K277Q (rnl2) or CircLig protocol with Mth K97A (mth). Despite both universal adaptor and enzyme being in greater than 3-fold molar excess of the fragment pool, Mth K97A did not successfully ligate all fragments (Figure 2). In addition, there was no improvement of ligation efficiency with increased incubation time. Ligated fragments were recovered and converted to cDNA libraries as described in the Methods. To reduce any potential confounding effect of PCR biases, all libraries were amplified under the same conditions prior to sequencing on an Ion Torrent PGM. Sequenced reads of the expected size were selected and trimmed to remove adaptor sequences and the (conserved) first 10 nt of the fragment (Additional file 1). Subsequent analysis was performed on the remaining 10 nt sequences.

**Figure 3:**
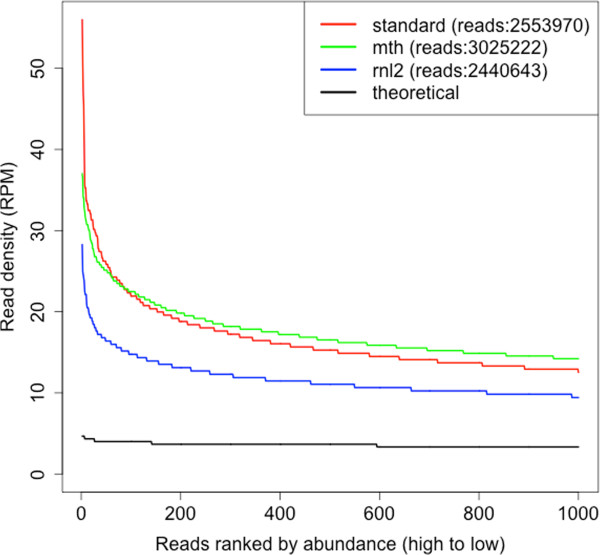
**Over-representation in sequencing libraries.** Sequencing libraries were generated from the partially degenerate RNA pool using either the standard protocol (standard), the CircLig protocol with trRnl2 K227Q (rnl2) or the CircLig protocol with Mth K97A (mth). The abundance (read density) of each unique sequence within the degenerate region was calculated as a ratio of the total read data (reads per million sequenced; RPM). The density of the 1000 most abundant sequences are presented for each library. The theoretical is X ~ Binominal(3x10^6^, 1/4^10^).

Degeneracy at 10 positions yields 1,048,576 (i.e. 4^10^) unique sequences at equimolar concentrations, giving a theoretical read distribution of X ~ Binomial(n,1/4^10^) where X is the number of times a particular sequence is observed in n sequenced reads. All library protocols resulted in read distributions that were over-dispersed relative to the theoretical (Figure 3 and Additional file 2). Goodness of fit comparisons between each distribution and the theoretical yielded the minimum computable p-value in the R software environment (discrete Kolmogorov –Smirnov test p < 2.2x10^-16^). The most abundant sequences were present at least 5 times more than would be expected in the absence of bias. However, the most abundant sequences from the trRnl2 K227Q CircLig library were approximately half as over-dispersed as those from the standard library and reached statistical significance (discrete Kolmogorov-Smirnov test, p < 2.2x10^-16^). Somewhat surprisingly, replacing trRnl2 K227Q with Mth K97A did not further reduce over-representation.

### Position specific biases

**Figure 4:**
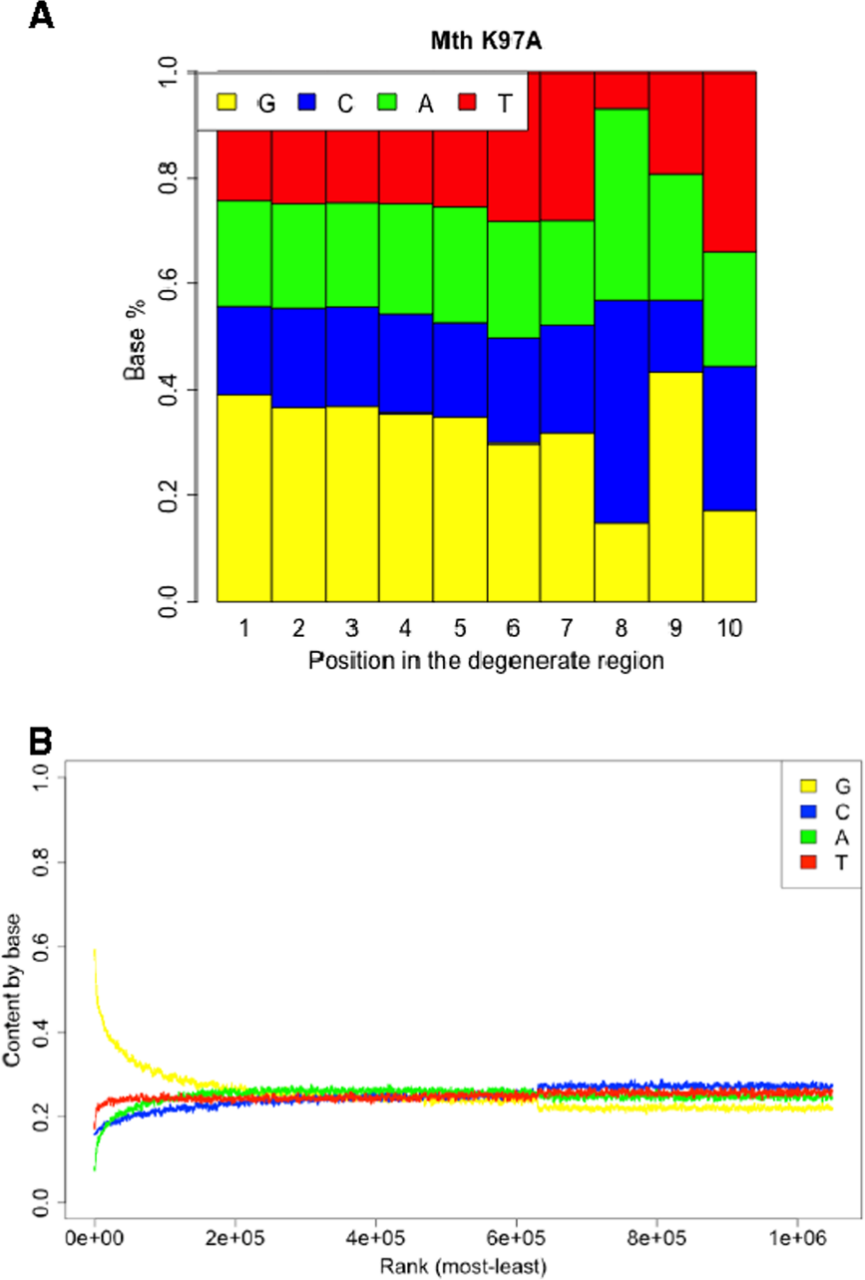
**Position specific bias associated with Mth K97A.** The abundance of each nucleotide at each position within the degenerate portion of the sequenced reads was calculated. **(A)** The use of Mth K97A in library construction was associated with pronounced over-representation of A and C at the 3^rd^ nucleotide from the ligation site. **(B)** Sequences were ranked based on abundance and the nucleotide content calculated across a sliding window of 1000 sequences.

To identify position specific biases associated with each protocol, the nucleotide content at each position within the degenerate region was calculated. Use of Mth K97A was associated with a strong preference for adenine and cytosine at the 3^rd^ nucleotide from the ligation site (Figure 4A). The other protocols did not result in position specific bias, however all libraries had a higher than expected guanidine content (Figure 4B and Additional file 3). Elevated G-content was only observed in the most abundant reads, suggesting this bias cannot be explained by incorrect formulation of the fragment pool.

### Correlation between predicted structure and over-representation

To assess the association between the secondary structure of the RNA fragments and co-folding with adaptor sequences, *in silico* folding experiments were performed under the conditions used for each library protocol. 

**Figure 5:**
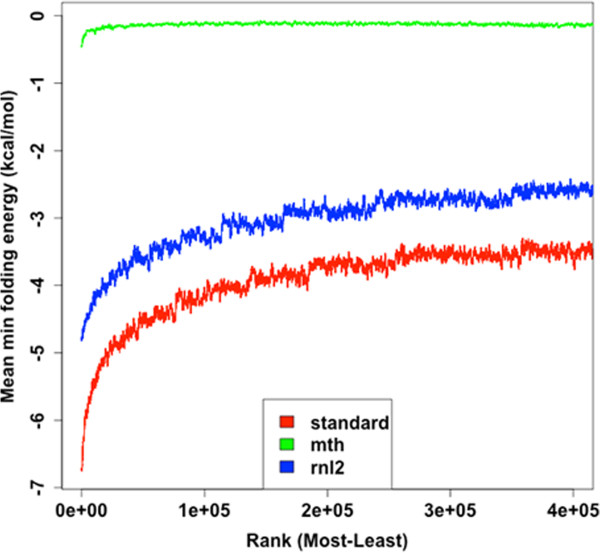
**Over-represented sequences are more likely to be structured.** The predicted folding energy for each unique sequence under the ligation reaction conditions was calculated. Sequences were ranked based on abundance and the mean folding energy calculated across a sliding window of 1000 sequences.

**Figure 6:**
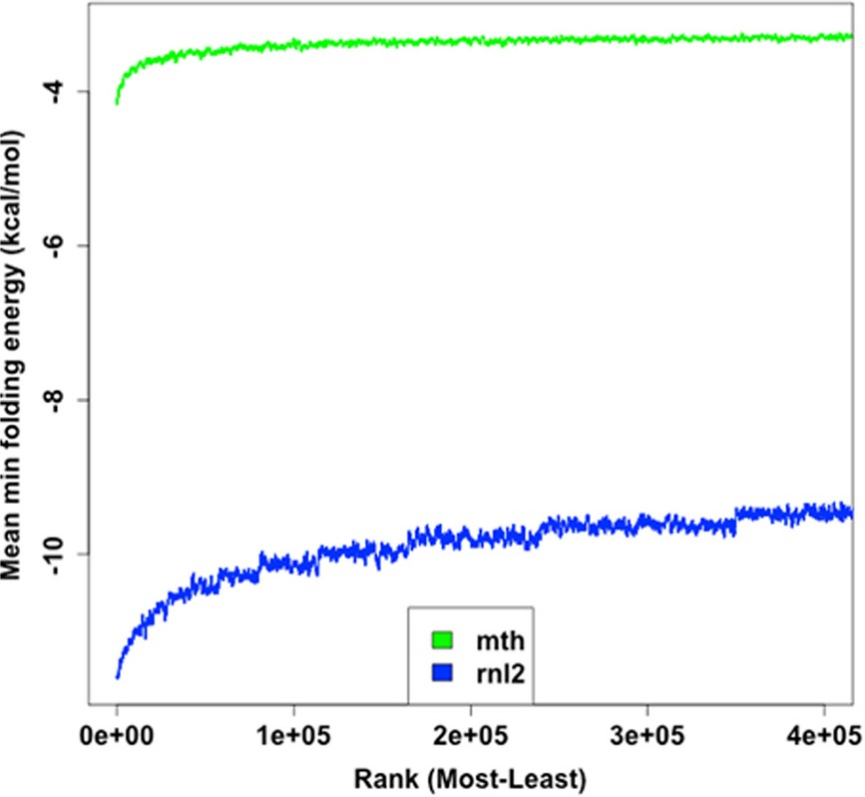
**Over-represented sequences are more likely to be predicted to co-fold with the adaptor.** The predicted folding energy between the adaptor sequence and each unique RNA fragment sequence was computed. Sequences were ranked based on abundance and the mean folding energy calculated across a sliding window of 1000 sequences.

Over-representation of fragments was correlated with degree of predicted secondary structure in all libraries (Figure 5). Duplex adaptors could not be modelled preventing assessment of co-folding in the standard protocol, but for the CircLig protocols (rnl2 and mth), over-represented fragments were more likely to co-fold with the adaptor (Figure 6).

Importantly, only the most over-represented fragments from the Mth library were associated with any secondary structure or co-folding ability suggesting that secondary structure is not a major source of bias when this protocol and enzyme are used.

## Discussion

Next-generation sequencing can be used for a range of genomic investigations. However, as with any technology, systemic biases affect the accuracy of sequencing data and thus the strength of conclusions that can be drawn. The ligation step in library generation has been shown to be a significant source of bias. We found that compared to the standard protocol the CircLig protocol with trRnl2 K227Q was associated with almost half the level of over-representation when a degenerate RNA pool was sequenced. Although the adaptor sequences for library generation differ between Life Technologies SOLiD and Ion Torrent platforms, the protocols are essentially similar. Therefore, using the correct adaptor sequences, the CircLig approach could be used for any Life Technologies platform and be expected to produce more representative libraries than the standard protocols.

Analysis of the sequencing data from both standard and CircLig protocols revealed the sequences that were over-represented were predicted to be more structured under the ligation conditions. This correlation may be causative as T4 RNA ligase 2 is involved in the repair of nicked dsRNA [12], but this paper does not seek to address this question directly. Instead we show that ligation with the thermostable ligase Mth K97A at temperatures not broadly permissive for secondary structure and co-folding does not reduce over-representation.

A substantial portion of the RNA pool could not be ligated using Mth K97A (Figure 2). The initial characterisation of the ligation efficiency of this enzyme was performed with one RNA sequence [11]. By using a partially degenerate fragment pool we are able to characterise the enzyme more fully and reveal the enzyme has a strong preference for A and C at the 3^rd^ nucleotide from the ligation site. Consistent with this, much higher ligation efficiencies were obtained when the enzyme was used by Zhelkovsky and Reynolds to ligate RNA with A at this position [11] than we observed with our partially degenerate RNA pool. While we cannot exclude the possibility that this is specific to the adaptor sequence used (rather than a general limitation of this enzyme), this bias does make Mth K97A inappropriate for use in existing sequencing protocols.

Surprisingly, the most abundant sequences in all libraries had a higher than expected G-content. It is unclear whether this is because each library protocol has a previously unknown bias in favour of G-rich sequences or if the bias is at a step common to all libraries. We suggest this may be an interesting avenue for further research.

## Conclusions

The CircLig protocol reduces, but does not abolish, bias associated with Ion Torrent PGM RNA-seq. Highly structured sequences are more likely to be over-represented in RNA-seq libraries, but this is not remedied by the use of the thermostable Mth K97A enzyme. Although the CircLig protocol does involve more hands-on time than the standard Life Technologies protocol, it offers superior accuracy and therefore we recommend it for sequencing on Life Technologies platforms.

## Methods

### Design of fragment pool

A random number generator ( http://www.random.org) was programmed to output values between 1 and 4 inclusive, with each digit corresponding to a different base. The program was run 10 times giving the following sequence: GCAGUUGCCA. An RNA fragment pool with this sequence followed by 10 degenerate nucleotides (5′ – GCAGUUGCCANNNNNNNNNN – 3′) was synthesised (ThermoScientific Molecular Biology). Fragments contained both 5′ phosphate and 3′ hydroxyl moieties necessary for ligation.

### Small RNA library construction

For standard libraries, ligation of 10 ng of RNA pool was performed with the Ion Total RNA-Seq kit v2 (Life Technologies), following the manufacturer’s instructions.

For the CircLig libraries, 40 ng RNA pool (~6 pmol) and 100 ng Universal Cloning Linker (~18 pmol; NEB) were denatured at 80°C for 2 min then placed immediately on ice. Ligation with 200 U T4 RNA Ligase 2 truncated K227Q mutant (trRnl2 K227Q; NEB) was performed in the presence of 1X RNA ligase buffer (NEB), 15% PEG8000, 20 U SuperaseIn (Ambion), at 25°C for 3 hr. Ligation with 20 pmol Mth K97A was performed in 1X NEB1 buffer and 20 U SuperaseIn, at 65°C for 1 hr, as described by Zhelkovsky and McReynolds (11). Reactions were terminated by heat inactivation at 90°C and RNA/DNA recovered by isopropanol precipitation with 1 μl GlycoBlue (Ambion) as a coprecipitant. Reaction mixtures were denatured at 80°C in an equal volume of formamide loading buffer (96% Formamide, 10 mM EDTA, 0.01% bromophenol blue, 0.01% xylene cyanol) and separated on a 15% TBE-Urea polyacrylamide gel (Invitrogen). 250 ng small RNA marker (Abnova), denatured the same way, was run in adjacent wells. Gels were stained with SybrGold (Invitrogen) and visualised using a Safe Imager (Invitrogen) to guide excision of the 37 nt band. RNA/DNA was recovered from the gel slice by overnight elution in 300 mM NaCl, 0.1% SDS at 4°C followed by isopropanol precipitation and resuspension in 10 μl nuclease-free water. 2 μl of 1.25 μM Ion Torrent compatible reverse transcription (RT) primer (5′-CGCCTTGGCC/Sp/CACTCA/Sp/CCTCTCTATGGGCAGTCGGTGATATCTATTGATGGTGCCTACAG – 3′ where Sp is an 18-atom hexa-ethyleneglycol spacer) was added to each sample, denatured at 80°C for 3 min then snap cooled on ice. Reverse transcription was performed using 200U Superscript III (Invitrogen) at 48°C for 30 min in 1X first strand buffer (Invitrogen), 0.5 mM dNTPs, 5 mM DTT and 20 U SuperaseIn. RNA was hydrolysed by incubation at 98°C for 20 min with 100 mM NaOH, 50 mM EDTA. cDNA was recovered by isopropanol precipitation and separated on a 6% TBE-Urea polyacrylamide gel as described above. The upper band (~80 bp) was excised, being careful to avoid the lower weight bands (unincorporated RT primer and RT primer dimers). cDNA was recovered from the gel slice and circularised with 100 U Circligase (Epicentre) in 1X Circligase buffer, 2.5 mM MnCl_2_, 0.5 mM ATP, at 60°C for 1 hr. Reactions were heat inactivated at 80°C and cDNA precipitated.

### Sequencing

All libraries were PCR amplified and sequenced using an Ion Torrent 318 chip as per manufacturer’s instructions, with the difference in library size in the CircLig protocol accounted for to ensure the same molarity of each library was used.

### Bioinformatic analysis

The PCR adaptors were removed from the reads using the standard Ion Torrent pipeline. Sequenced reads were then filtered based on their size: 18 nt to 22 nt inclusive for reads from the standard protocol, 40 nt to 44 nt inclusive for CircLig based protocols. A custom script using Smith-Waterman alignments, with identity >0.7, was used to remove the linker sequence from the 3′ end of the alternate protocol reads, and to remove the 10 nt non-degenerate region from all reads.

Read sets were analysed to determine how many times each particular sequence was observed. A theoretical dataset corresponding to the total number of sequenced reads from each library was constructed, i.e. a distribution X ~ Binomial(n, 1/4^10^), where n was the total number of reads. Goodness of fit testing was performed using a discrete Kolmogorov –Smirnov test from the R Package ‘Matching’ with 500 iterations of bootstrapping.

The predicted structure of each read, including the 10 nt non-degenerate region, was produced using RNAfold from the ViennaRNA Package [13], and the minimum free energies were plotted against the reads ranked from most to least abundant, using the mean minimum free energy over a sliding 1000-read window. Co-folding structures between the fragment (GCAGUUGCCANNNNNNNNNN) and the linking sequence used in the alternative protocols (CUGUAGGCACCAUCAAU) were also predicted using RNAcofold from the ViennaRNA Package, and plotted in the same way. Predictions were made at 25°C for the rnl2 dataset, at 65°C for the mth dataset, and at 16°C for the standard protocol dataset. All other parameters were left as defaults. Plots were produced using R ( http://www.R-project.org). Data are available in the ArrayExpress database ( http://www.ebi.ac.uk/arrayexpress) under accession number E-MTAB-2566.

## Electronic supplementary material

Additional file 1: **Trimming of sequenced data.** The length of reads from the standard **(A)**, rnl2 **(B)** and mth **(C)** libraries before and after length filtering and then trimming adaptors and the non-degenerate region. (PDF 150 KB)

Additional file 2: **Comparison of over-representation using equal total read numbers.** The first 1900000 reads from each library were taken. The abundance (read density) of each unique sequence within the degenerate region was calculated as a ratio of the total read data (reads per million sequenced; RPM) as per Figure 3. (TIFF 1 MB)

Additional file 3: **Nucleotide content of read data.** Left: The abundance of each nucleotide at each position within the degenerate portion of the sequenced reads for standard (top) and rnl2 (bottom). Right: The corresponding nucleotide content for the sequenced reads, ranked from most to least abundant, calculated across a sliding window of 1000 sequences. (PDF 195 KB)
