# Supplementary figures and images for: Evaluating bias-reducing protocols for RNA sequencing library preparation
Source: BMC Genomics. 2014 Jul 7;15(1):569. doi: 10.1186/1471-2164-15-569 (PMC4117970; doi:10.1186/1471-2164-15-569)

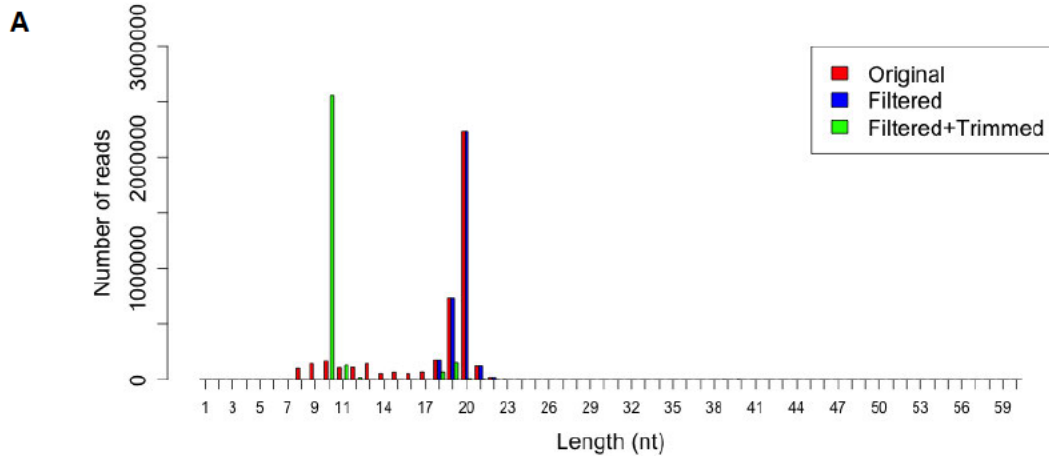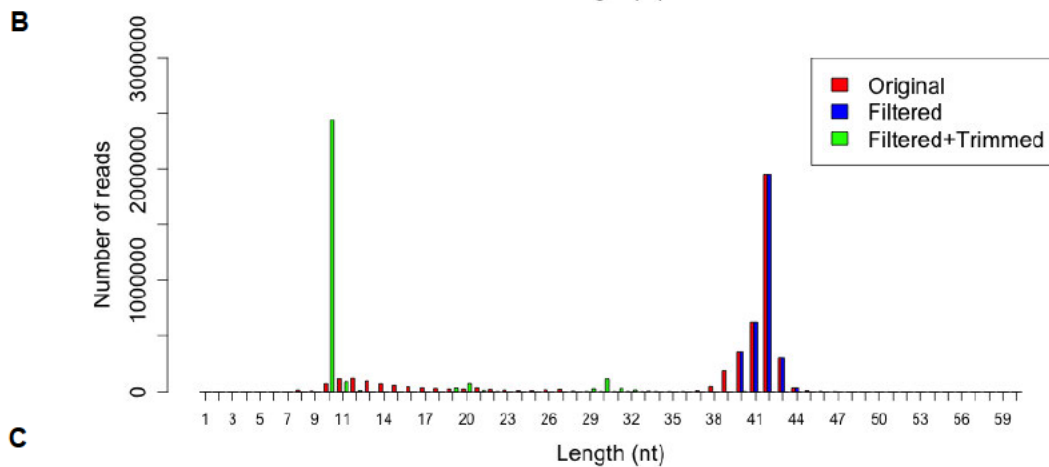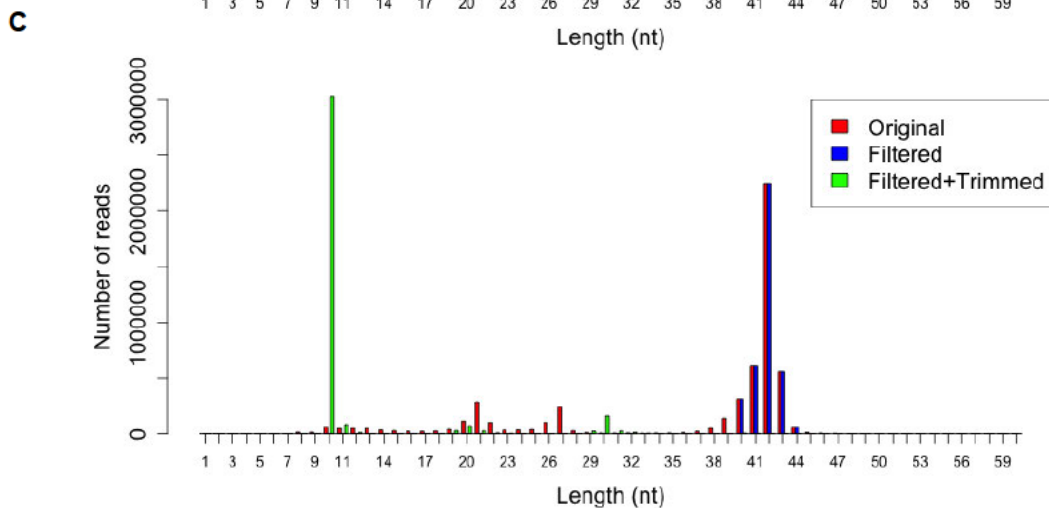

Supplement: Supplementary file 1 — Additional file 1: Trimming of sequenced data. The length of reads from the standard (A), rnl2 (B) and mth (C) libraries before and after length filtering and then trimming adaptors and the non-degenerate region. (PDF 150 KB) [file 12864_2014_6289_MOESM1_ESM.pdf]

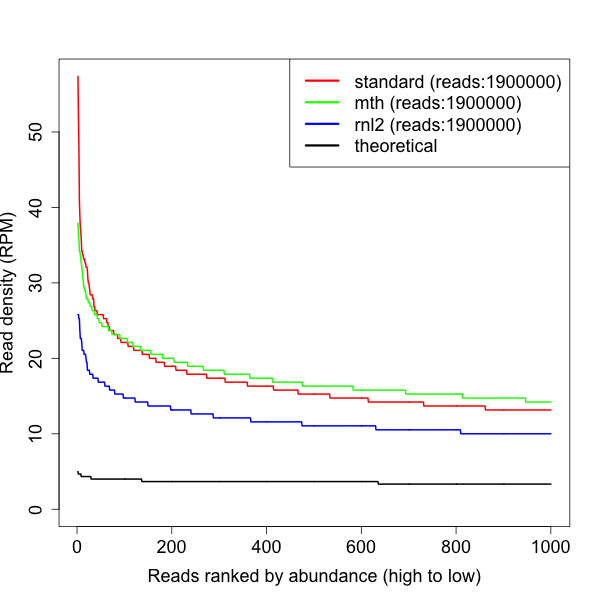

Supplement: Supplementary file 2 — Additional file 2: Comparison of over-representation using equal total read numbers. The first 1900000 reads from each library were taken. The abundance (read density) of each unique sequence within the degenerate region was calculated as a ratio of the total read data (reads per million sequenced; RPM) as per Figure 3. (TIFF 1 MB) [file 12864_2014_6289_MOESM2_ESM.tiff]

Standard

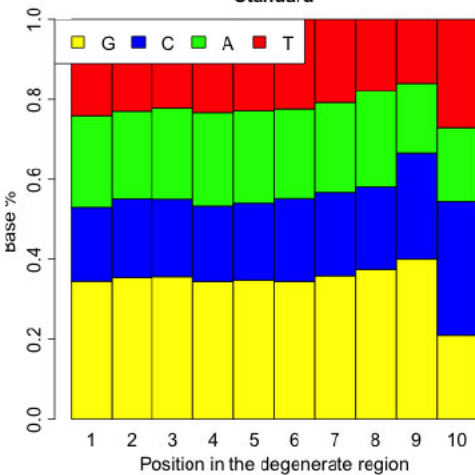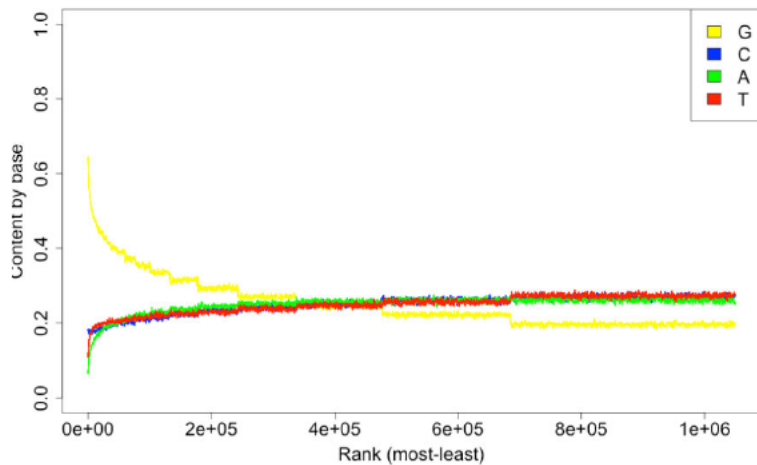

trRnl2 K227Q

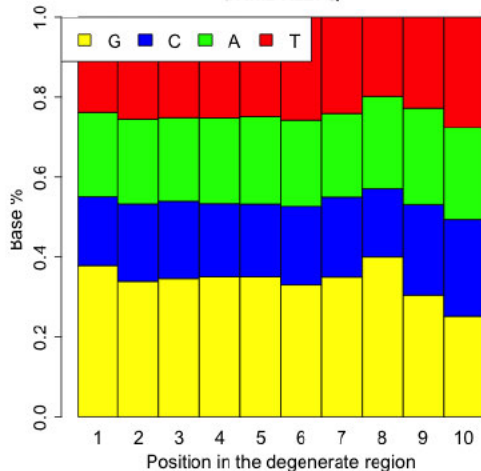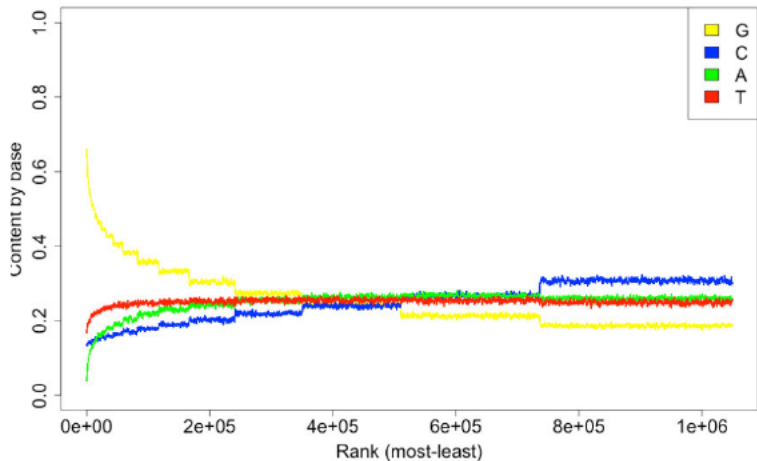

Supplement: Supplementary file 3 — Additional file 3: Nucleotide content of read data. Left: The abundance of each nucleotide at each position within the degenerate portion of the sequenced reads for standard (top) and rnl2 (bottom). Right: The corresponding nucleotide content for the sequenced reads, ranked from most to least abundant, calculated across a sliding window of 1000 sequences. (PDF 195 KB) [file 12864_2014_6289_MOESM3_ESM.pdf]
